# Dung beetles increase plant growth: a meta-analysis

**DOI:** 10.1098/rspb.2023.2885

**Published:** 2024-03-20

**Authors:** Daniel J. Anderson, Jacob D. Berson, Raphael K. Didham, Leigh W. Simmons, Theodore A. Evans

**Affiliations:** ^1^ School of Biological Sciences, The University of Western Australia, Perth, Western Australia, Australia; ^2^ Centre for Evolutionary Biology, School of Biological Sciences, The University of Western Australia, Perth, Western Australia, Australia; ^3^ Centre for Environment and Life Sciences, CSIRO Health and Biosecurity, Floreat, Western Australia, Australia

**Keywords:** coprophagy, dung burial, ecosystem function, ecosystem service, Scarabaeoidea, systematic review

## Abstract

The ecosystem services provided by dung beetles are well known and valued. Dung beetles bury dung for feeding and breeding, and it is generally thought that the process of burying dung increases nutrient uptake by plant roots, which promotes plant growth. Many studies have tested the effects of dung beetles on plant growth, but there has been no quantitative synthesis of these studies. Here we use a multi-level meta-analysis to estimate the average effect of dung beetles on plant growth and investigate factors that moderate this effect. We identified 28 publications that investigated dung beetle effects on plant growth. Of these, 24 contained the minimum quantitative data necessary to include in a meta-analysis. Overall, we found that dung beetles increased plant growth by 17%; the 95% CI for possible values for the true increase in plant growth that were most compatible with our data, given our statistical model, ranged from 1% to 35%. We found evidence that the dung beetle–plant growth relationship is influenced by the plant measurement type and the number of beetles accessing the dung. However, beetles did not increase plant growth in all quantitative trials, as individual effect sizes ranged from −72% to 806%, suggesting important context-dependence in the provision of ecosystem services.

## 1. Introduction

Humanity depends upon natural ecosystem functions [1,2]; the bio-geochemical processes that sustain ecosystem productivity [3]. Ecosystem functions can provide goods and services that directly and indirectly benefit humans, such as food, fibre, clean air, water and soil [1]. Pollination of plants for example, underpins 35% of global crop production volume, valued at USD 235–577 billion [4,5], while predation of herbivorous species is valued at USD 417 billion [6]. Despite their critical importance, the provision of ecosystem services will be substantially diminished by the current human-induced mass extinction [7–10]. Preserving ecosystem services will require interventionist policies that incorporate natural capital into resource and land-use decisions [11,12]. However, such policies require robust estimates of the ecosystem services provided by different organisms [1,13].

Dung removal and burial, the primary ecological processes performed by dung beetles, underpin some of the most widely known and highly valued ecosystem services provided by insects [14–16]. Dung beetles are considered to be ecosystem engineers because of the multiple ecosystem services that arise from the primary activity of dung removal and burial for feeding and breeding. They filter the liquid component from the dung as food [17], which then restricts the growth of nuisance dung-breeding flies [18]. When burying dung for breeding, beetle tunnelling increases aeration and water infiltration into the soil profile [19–22] and creates preferential paths for plant root growth [23]. Moreover, the burial of dung within breeding tunnels increases nutrient availability in the soil [24–26]. Additionally, beetle tunnelling can promote seedling establishment by relocating seeds to optimal germination depths. Seeds can be relocated downwards into the soil (through dung burial) [27], or upwards within the soil (through soil exhumation) [28,29]. Together, these multiple ecosystem functions can increase nutrient uptake by plant roots, thereby increasing plant biomass and photosynthetic capability [19,30–32].

Although it is generally thought that dung beetles increase plant growth [16], their influence can be minimal or even negative [33–35]. For example, the influence of beetle tunnelling can vary with climate [35]. In cooler climates beetle tunnelling might increase soil water infiltration and promote plant growth, but in warmer climates beetle tunnelling might increase soil drying by evaporation and reduce plant growth. Different plant species can also respond differently to dung beetle activity. For example, dung burial that benefits light-demanding temperate grassland species, might hinder shade-tolerant rainforest species [34]. Alternatively, nutrients from buried dung might be absorbed immediately by a mesotrophic plant and slowly by an oligotrophic plant [32].

There are many factors that might influence the dung beetle–plant growth relationship, including beetle species diversity, dung manipulation (functional group), or climate and soil conditions. There are approximately 6000 dung beetle species worldwide [36], and plant growth can be influenced more by the species composition of dung beetles, than the actions of any one species [37]. Dung beetles also manipulate dung in different ways; some bury (tunnellers), some relocate and bury (rollers), and some live within the dung (dwellers) [38]. These different ways of manipulating dung (often referred to as ‘functional groups’) can influence plant growth [32]. Moreover, the influence of dung beetles on plant growth can be greater in drier soil [31], in less-compacted soil [39], in areas with reduced precipitation [19], when burying dung from grass-fed livestock [40], or in the presence of plants with higher N uptake [32]. These factors suggest that the dung beetle–plant growth relationship might be more nuanced than considered in many previous studies, and contingent on the local environmental and/or experimental conditions [33].

Given the potential for variation in the dung beetle–plant growth relationship across environmental conditions, individual dung beetle species traits and community composition, quantifying the overall impact of dung beetles on plant growth poses a significant challenge. Modern meta-analytical techniques can be used to compile data from multiple sources to generate an estimate of the overall effect size, simultaneously examining the factors contributing to variation in plant growth across studies [41]. Here, we conduct a systematic review and meta-analysis to (a) quantify the effect that dung beetles have on plant growth, (b) investigate the possible factors that influence this effect and (c) investigate the sources of variation in plant growth across studies.

## 2. Methods

### (a) Systematic review

**Figure 1.**
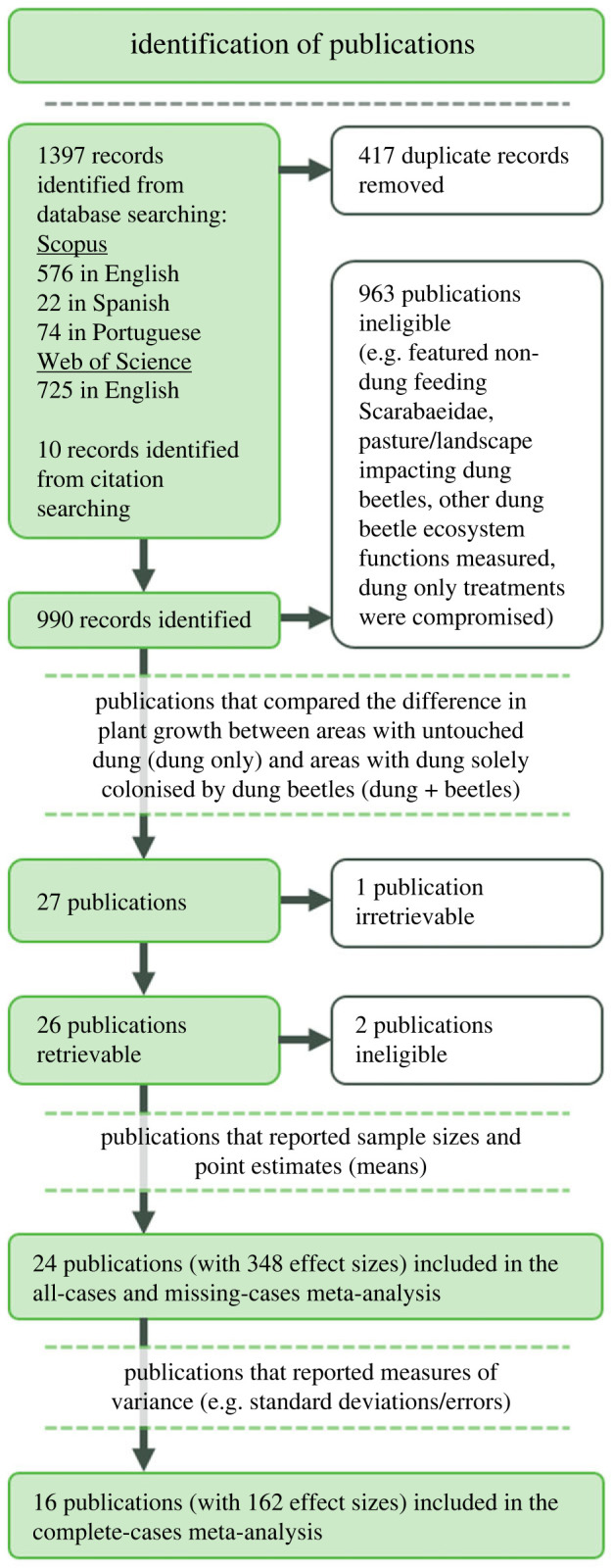
A systematic review following the PRISMA 2020 method [42]. We identified 1397 publications from our search. Twenty-four of these publications had sufficient information to calculate a pooled coefficient of variation and were included in the meta-analysis. The pooled coefficient of variation was used; on all individual effect sizes with both known and unknown measures of variance (the all-cases approach, which was our primary approach); and only on individual effect sizes with unknown measures of variance (the missing-cases approach). Of the 348 individual effect sizes, those with an unknown measure of variance were then excluded from the complete-cases meta-analysis.

To identify suitable publications for our meta-analysis, we conducted a systematic review, following the PRISMA 2020 method [42] and the recommendations of Nakagawa *et al*. [43] (figure 1). On 3 November 2023, we conducted a literature search on the *Scopus* and *Web of Science* databases using the following search terms, repeated in English, Spanish and Portuguese, without filters or limitations: ((‘dung beetle*’ OR scarabaei*) AND (herb* OR pasture OR plant* OR primary OR seedling*) AND (growth OR increase* OR product* OR yield)). We based these search terms on relevant publications already known to us. Search term translations into Spanish and Portuguese are detailed in the electronic supplementary material.

We identified 1397 publications from our search, and only those publications that met the following criteria were included in our meta-analysis:

1) studies that compared the difference in plant growth between areas with untouched dung (dung only) and areas with dung colonised solely by dung beetles (dung + beetles); and
2) studies in which the results had a sample size, point estimates (means) of plant growth in the ‘dung only’ and ‘dung + beetles’ treatments, and where possible, a measure of precision of the means (standard deviation/error).

The 24 publications that could be included in our meta-analysis were conducted in 14 countries from 1970–2023 (electronic supplementary material, figure S1). In total, at least 33 dung beetle species were studied (some studies did not determine species identities), using five types of mammalian dung. The dung beetles influenced agricultural and non-agricultural plants and plant communities, which were grown in various soil types and climates for periods ranging from 7 to 468 days.

### (b) Effect size calculation

We used the natural log of the response ratio (lnRR) as our measure of effect size, which is the log proportional change in yield between plants with dung + beetles and plants with dung only [44]. The lnRR value can be back-transformed to the proportionate change of plant growth using exp(lnRR) 1. To calculate an effect size for plant growth, we included height or weight measurements from the shoot, root or total plant. Height and weight measurements are comparable in this analysis as the resulting effect size is unitless [43]. Where study outcomes were presented in figures, we extracted values using the software DataThief [45].

In all meta-analyses of disparate studies there will be missing cases in some data fields, notably measures of variance [46]. When studies lacked any of the plant growth measurements needed to calculate an overall effect size (sample size, mean and standard deviation/error), we contacted authors for raw data. For those studies that reported a sample size, mean and a standard deviation/error, we were able to calculate a coefficient of variation with which to calculate an overall effect size [47]. We then used these known coefficient of variation values to calculate a pooled coefficient of variation and used that single value as a measure of variance for all individual effect sizes (regardless of a known or unknown standard deviations/errors). Using a pooled coefficient of variation for all effect sizes is known as an ‘all-cases’ approach, and recommended by Nakagawa *et al*. [47]. We used the code provided by Nakagawa *et al*. [47] in their func.R script and the metafor package [48] in R (v4.3.2) [49] to calculate effect sizes and conduct a multi-level meta-analysis.

### (c) Multi-level meta-analysis

The identified publications contained many multiple-treatment studies that assessed plant growth under different conditions, such as comparing how dung beetles influence plant growth among different soil types, temperatures or years. The 24 studies we assessed contained 348 effect sizes. When a study contains multiple effect sizes they can potentially be correlated, and these correlations can inflate the precision of the estimated overall effect size if they are not accounted for in the meta-analysis [50]. Therefore, to estimate the overall influence of dung beetles on plant growth, we used a multi-level meta-analytic model which takes into account potential correlations within studies [43]. We used the heterogeneity statistic *I*^2^ to calculate how much variation is attributable to each of the multiple levels within the meta-analysis (i.e. overall, between studies and between effect sizes) [51].

There were numerous types of correlation between the effect sizes in the publications we identified. For example, some studies included a shared control, where multiple dung + beetles treatments were compared to the same dung only treatment (e.g. [52]). Some studies had shared measurements, where more than one measurement/response was taken from the same plant (e.g. [19]). Within-study temporal correlation was also identified, where plants were measured at multiple timepoints (e.g. [32]). We took two steps to address correlation within studies. To correct for a shared control within a study, we divided the sample size of the shared (dung only) control by the number of dung + beetles treatments it was compared with, then calculated the precision of the effect size [53]. To correct for shared measurements and within-study temporal correlation, we created a variance–covariance (vcov) matrix that assigned a level of correlation between those effect sizes with shared measurements and/or temporal correlation [54]. In meta-analyses like this, where the degree of correlation among the effect sizes is unknown, a correlation value is assigned (i.e. ranging from no correlation to perfect correlation). We chose 0.5 as the correlation value, which is a common approach [55], and a value that reflected the degree of correlation among our effect sizes. We then compared bias-corrected Akaike information criterion (AICc) values of multi-level meta-analytic models both with and without a vcov matrix (i.e. comparing a model that assumes correlation between effect sizes within studies, to a model that does not).

### (d) Sensitivity tests

We conducted two sensitivity tests to evaluate the robustness of our overall effect size estimate to the choice of correlation value (0.5) between effect sizes, and the use of a pooled coefficient of variation to all effect sizes. Firstly, we varied the assigned correlation value between those effect sizes with shared measurements and/or temporal correlation. We chose a correlation value of 0.5 to calculate an overall effect size, then as a sensitivity test, we varied this correlation value from 0.1 (low correlation) and 0.9 (high correlation) to determine its potential influence. Secondly, we changed the coefficients of variation that were used to calculate an overall effect size. We used the recommended all-cases approach (using a single, pooled coefficient of variation as a measure of variance for all individual effect sizes). As a sensitivity test, we also used the ‘missing-cases’ approach and the ‘complete-cases’ approach [47]. The missing-cases approach uses a coefficient of variation for individual effect sizes with known standard deviations/errors, and a pooled coefficient of variation for individual effect sizes with unknown standard deviations/errors. The complete cases approach uses a coefficient of variation for individual effect sizes with known standard deviations/errors, and excludes individual effect sizes with unknown standard deviations/errors.

### (e) Moderators

To examine the variation in effect sizes, we used multi-level meta-regression models to assess how moderators (fixed effects) influenced the direction and magnitude of dung beetle effects on plant growth. For each effect size we recorded the following moderating variables where possible: (1) beetle functional group (tunneller, roller, dweller or multiple), (2) number of beetles, (3) species richness of beetles, (4) dung quantity (g) (when dung quantity was recorded in litres, we converted it to grams using 1 l = 877 g) [35,56], (5) dung type, (6) surface area of enclosure and (7) plant measurement type.

Most of the moderators contained missing values, making it difficult to analyse them in combination, so we assessed their influence in two ways. First, we tested moderators individually, adding each to the null model and then comparing it against the original null model with a likelihood ratio test (based on a *p*-value of 0.05), which is a common approach in meta-analyses [57]. We used marginal *R*^2^ as a summarizing statistic for the variance explained by each moderator [58]. Second, as there might have been covariance among moderators, we tested moderators in combination. We used the mice package to impute missing moderator values [59,60], then used metafor to build a global model that included all seven moderators. We then calculated the AICc for all combinations of fixed effects using the ‘dredge’ function from the MuMIn package [61]. The model with the lowest AICc value, and those within two units, were deemed the most parsimonious. We used the sum of model weights to determine the relative important of each moderator.

We visualized the results from our models using orchard plots [62]. An orchard plot contains a confidence interval (CI) and prediction interval (PI) around an overall effect size estimate. The confidence interval represents the range within which the overall effect size of the meta-analysis would likely occur. A prediction interval is the range within which the effect size of a new study outcome would be predicted to lie, given this new outcome was selected at random from the same population of studies as those already included in the meta-analysis [63]. Each point represents the scaled individual effect size of a study outcome. The position of each point along the *x*-axis is determined by its effect size. Points along the *y*-axis are scattered to both reduce overplotting and help visualize point density.

### (f) Publication bias

Studies that have statistically significant, positive results are more likely to be published than studies that do not [64]. This can mean that a meta-analysis will misrepresent the true effect size, by producing biased model estimates. To test for publication bias within a meta-analysis, Nakagawa *et al*. [43] recommended using at least two different methods. We assessed publication bias visually, by plotting effect sizes against the inverse of sample standard error as a measure of uncertainty (precision; 1/SE) in a funnel plot [65]. We also assessed publication bias statistically, by using a multi-level model version of Egger's regression, which includes sampling standard error as a moderator [57]. If Egger's regression results show a significant effect of sampling standard error, it suggests the presence of publication bias.

The supplementary material contains an annotated R code of our full analysis. When reporting our results we use the language of statistical ‘clarity’, as recommended by Dushoff *et al*. [66].

## 3. Results

**Figure 2.**
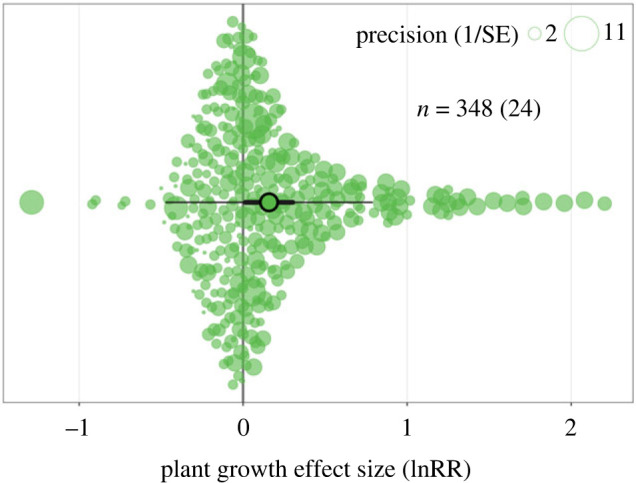
Dung beetles increase plant growth. Orchard plot of the 348 effect sizes (natural log of the response ratio, lnRR) taken from the 24 identified publications. The effect size points are scaled according to study variance (precision). The plot contains a confidence interval (thick, horizontal; CI 1% to 35%) and a prediction interval (thin, horizontal; PI −37% to 119%) around an overall mean effect size estimate (17%).

From the multi-level meta-analysis on 348 effect sizes contained within 24 eligible studies, a model estimate is that dung beetle activity increases plant growth by 17% on average (95% CI 1% to 35%; figure 2). However, the prediction interval (95% PI −37% to 119%) shows that the predicted outcomes for future studies include negative effects of dung beetles on plant growth. The total heterogeneity in the data was low ($I_{\mathrm{Total}}^{2}=33.07$), with variance at the study level accounting for 87% of the variance ($I_{\mathrm{Level}\;2:\mathrm{StudyID}}^{2}=28.9$) and within study level variance 13% of the total ($I_{\mathrm{Level}\;3:\mathrm{AccessionID}}^{2}=4.2$), respectively.

### (a) Sensitivity tests

Sensitivity tests of our overall effect size estimate showed that qualitatively similar results were obtained irrespective of either the correlation value used, or the approach taken to missing estimates of effect size variance. The overall effect size (17%; CI 1% to 35%) was similar to estimates assuming low correlation (17%; CI 1% to 35%; electronic supplementary material, table S1) and high correlation (16%; CI 1% to 35%). Additionally, the overall effect size remained positive when calculated with the missing-cases approach (18%; CI 5% to 33%; electronic supplementary material, figure S2, table S2) and complete-cases approach (11%; CI 4% to 17%; electronic supplementary material, figure S2, table S3).

### (b) Moderators

**Figure 3.**
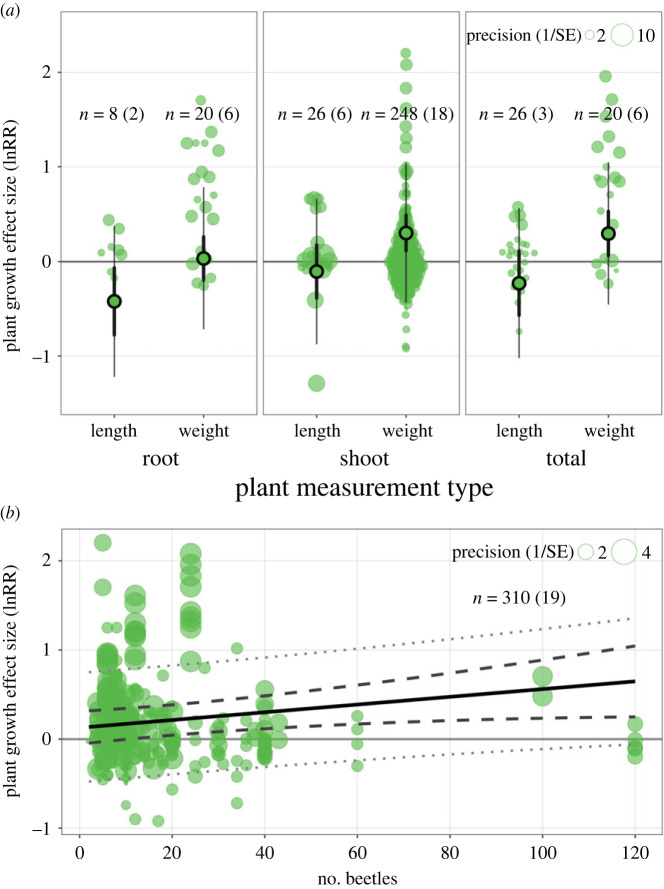
Moderator effects on the relationship between dung beetles and plant growth. (*a*) Orchard plot of the effect of plant measurement type on effect size estimates. The plot shows 348 effect sizes (natural log of the response ratio) taken from 24 publications that reported plant measurement type. The effect size points are scaled according to study variance (precision). Each plant measurement type contains a confidence interval (thick, vertical) and prediction interval (thin, vertical) around an overall effect size estimate. (*b*) Orchard plot of the effect of the number of beetles on effect size. The plot shows 310 effect sizes (natural log of the response ratio) taken from 19 publications that reported the number of beetles. The effect size points are scaled according to study variance (precision). The model-predicted relationship is shown by the solid black line, the 95% confidence interval by the dashed lines, and the 95% prediction interval by the dotted lines. The solid grey line indicates no effect of dung beetles on plant growth.

Assessing moderators individually within multi-level meta-regression models identified plant measurement type as being influential to the dung beetle–plant growth relationship ($R_{\mathrm{marginal}}^{2}=0.231$, $\chi_{8}^{2}=23.07$, *p* < 0.001; figure 3*a*, electronic supplementary material, table S3). In particular, the effect sizes calculated from shoot weight (35%; CI 12% to 63%) and total weight (35%; CI 7% to 70%) were highest, and those from root length (−34%; CI −54% to −6%) the lowest. The number of beetles positively influenced the dung beetle–plant growth relationship ($R_{\mathrm{marginal}}^{2}=0.065$, $\chi_{4}^{2}=5.5$, *p* < 0.05; figure 3*b*, electronic supplementary material, table S3). For example, the overall estimate of plant growth roughly quadrupled when the number of beetles increased from 10 (19%; CI 0% to 42%) to 100 (75%; CI 27% to 144%).

The effects that beetle functional group ($R_{\mathrm{marginal}}^{2}=0.056$, $\chi_{6}^{2}=1.18$, *p* = 0.758), species richness ($R_{\mathrm{marginal}}^{2}=0.002$, $\chi_{4}^{2}=0.21$, *p* = 0.65), dung quantity ($R_{\mathrm{marginal}}^{2}=0.053$, $\chi_{4}^{2}=2.21$, *p* = 0.137), dung type ($R_{\mathrm{marginal}}^{2}=0.059$, $\chi_{7}^{2}=4.49$, *p* = 0.344), and the surface area of enclosure ($R_{\mathrm{marginal}}^{2}=0.112$, $\chi_{4}^{2}=1.99$, *p* = 0.158) had on the dung beetle–plant growth relationship were unclear. None of these moderator variables were statistically significant. Assessing moderators in combination (using imputed values for missing moderator values) indicated that the dung beetle–plant growth relationship is influenced by plant measurement type and the number of beetles ($R_{\mathrm{marginal}}^{2}=0.248$, $\chi_{19}^{2}=31.56$, *p* < 0.05; electronic supplementary material, table S4). Plant measurement type had the highest relative importance value for plant growth among the moderators (1.00; electronic supplementary material, table S5), followed by the number of beetles (0.65).

### (c) Publication bias

**Figure 4.**
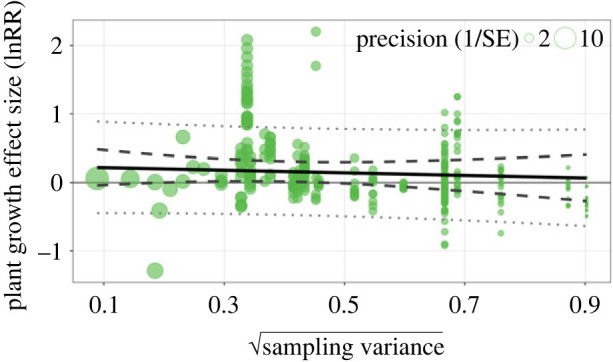
No statistical evidence of publication bias using Egger's regression. The sampling standard error of the Egger's regression is shown by the solid black line, the 95% confidence interval by the dashed lines, and the 95% prediction interval by the dotted lines. The solid grey line indicates no publication bias within the analysis. The effect size points are scaled according to study variance (precision).

A relationship between plant growth effect sizes and sampling standard error in Egger's regression was not statistically significant (−0.19; CI −0.82 to 0.44; figure 4), indicating no publication bias within the meta-analysis. When accounting for moderators, most of the data points in the funnel plots were within the pseudo-confidence region around their estimated means (electronic supplementary material, figures S4–S6).

## 4. Discussion

The ecosystem services that humanity depends upon are in decline [2]. Preserving ecosystem services will require robust estimates of their value, which can inform resource and land-use decisions [1,11–13]. The ecosystem services provided by dung beetles are well recognized, yet despite the widely held perception that dung beetles improve plant growth, there is surprisingly little quantitative data on this relationship. Of the 27 studies that appropriately assessed dung beetle influence on plant growth, only 24 reported their results to the standard required for inclusion in our meta-analysis. Our multi-level meta-analysis estimates that dung beetles increase plant growth by 17% on average (CI 1% to 35%). We found that the dung beetle–plant growth relationship was influenced by the plant measurement type, with weight measurements providing higher effect sizes than length measurements. Additionally, we found that the dung beetle–plant growth relationship was positively influenced by the number of beetles, with more beetles providing greater effect sizes.

The magnitude of the dung beetle effect on plant growth is comparable to the ecosystem services provided by other organisms that have been elucidated by meta-analyses. For example, van Groenigen *et al*. [67] estimate that (realistic densities of) earthworms increase crop yield by 10% to 21%. Some birds predate invertebrate herbivores, which indirectly preserves plant biomass. Mäntylä *et al*. [68] saw that plants within bird exclosures had 5% to 24% lower biomass than plants accessible to insectivorous birds (across a range of habitats). The presence of pollinators was seen to increase sunflower yield by up to 25% [69], while in the absence of pollinators reduced the yield of faba bean by 21% to 43% [57].

The effect that dung beetles had on plant growth varied among studies (−72% to 806%), so we assessed how moderators influenced the magnitude of plant growth. We saw that plant measurement type was an influential factor, with the effect sizes of plant weights generally being greater than plant lengths. Dung burial can increase the amount of N available to plants [32], allowing them to produce more mesophyll cells and hence thicker leaves with greater photosynthetic capability [30,39]. Additionally, the increased nutrient availability from dung burial might have caused a proliferation of root hairs, which would allow for greater nutrient uptake and negate investment in longer plant roots.

We saw that the number of beetles was also an influential factor, as plant growth was greater when more beetles accessed dung. Dung beetle abundance can correlate positively with dung removal [70,71], which could increase the amount of N available to plants [32] and lead to greater plant growth [33].

Unexpectedly, the influence of other components of biodiversity (species richness or functional group) on the dung beetle–plant growth relationship were not supported statistically. We were unable to assess the role of species evenness, as there was limited variation in evenness values among studies. Experimentally manipulating biodiversity is difficult, as creating a range of biodiversity values can require populations that are unrealistic to assemble [72]. We expected species richness and functional group to influence the magnitude of plant growth, as they can influence dung removal [56,70,71]. Individual studies saw that plant growth can be influenced by dung beetle richness [37] and functional group [32], but we saw no statistical evidence in our meta-analysis that species richness or functional group were influential. One reason for this might be that the effects were masked in our meta-analyses by the considerable variation in experimental design among studies.

Most of the variance in plant growth was due to the variance among studies ($I_{\mathrm{Level}\;2:\mathrm{StudyID}}^{2}=87\text{\%}$). The studies encompass a range of experimental conditions, with some being unfavourable to beetles and plants. The 24 studies included 14 countries, five dung types, at least 33 beetle species, and agricultural and non-agricultural plant species. Treatments within studies manipulated soil type [31], pasture management style [39] and plant measurement time [32,73–75]. Some studies included perturbances such as increased temperature [35], water reduction [19] and anthelmintic exposure [37]. This variation in experimental conditions and favourability is beneficial, as it makes the overall estimate more credible, and supports our general understanding that dung beetles increase plant growth [16].

The high variance among studies might also be due to avoidable variation in experiment design among studies. To limit variation among future studies, we suggest several protocols that should be standardized in dung beetle research: (1) unless specifically testing for the effect of anthelmintics on dung beetle activity, dung should be free of anthelmintics, which can be lethal or sub-lethal to dung beetles [76]; (2) unwanted resident coprophages should be removed by freezing dung prior to application, with 48 hr being a commonly used minimum duration; (3) variation in dung quality across treatments should be reduced by homogenizing dung before application; (4) mesocosms should be partially buried (and include a subterranean barrier if possible) to prevent incursion of unwanted coprophages [77]; and (5) to account for the excavated soil introduced into the dung by tunnelling beetles, dung samples should be ashed following beetle activity, best achieved by grinding the dried dung into a powder (granules less than 2 mm) and heating for two hours at 550°C [78].

In conclusion, we have used a systematic review to identify available studies of dung beetle effects on plant growth. We retrieved 26 studies, two of these saw that dung beetles increased plant growth, but failed to report a sample size and mean with which to calculate an overall effect size [26,40]. We used the remaining 24 studies in a multi-level meta-analysis to quantify the overall effect of dung beetles on plant growth, and explore how experimental and biological factors explain the variation among studies. Our meta-analysis and sensitivity tests indicated that dung beetles do increase plant growth. Our findings suggest that the magnitude of the dung beetle effect on plant growth is best explained by plant measurement type and the number of beetles. Our analysis indicated that further research could help clarify the influence of the other moderators, such as species richness and beetle functional group. Individual studies saw that beetle richness and functional group can influence plant growth, but this was not apparent in our overall synthesis. Therefore, we recommend that future research investigate how dung beetle biodiversity mediates the ecosystem services they provide, particularly species evenness. Future research should also implement our suggested experiment protocol to limit unwanted variation among dung beetle studies.

## Data Availability

The dataset and R code supporting this article have been provided as electronic supplementary material. Data are available from the Dryad Digital Repository: https://doi.org/doi:10.5061/dryad.pg4f4qrx3 [79]. The R code is provided in the electronic supplementary material [80].
